# First Described Case of Group B* Streptococcus* Pelvic Abscess in a Patient with No Medical Comorbidities

**DOI:** 10.1155/2016/3724706

**Published:** 2016-07-27

**Authors:** Paul Tyan, Elias Abi-Khalil, Karthik Dwarki, Gaby Moawad

**Affiliations:** ^1^Department of Obstetrics and Gynecology, George Washington University, Washington, DC 20037, USA; ^2^George Washington University School of Medicine & Health Sciences, Washington, DC 20037, USA

## Abstract

*Background*. Group B* Streptococcus* is an organism that commonly infects a wide range of hosts including infants in the first week of life, pregnant women, and older age adults as well as adults with underlying medical comorbidities.* Case*. Large pelvic abscess in a nonpregnant patient found to be caused by Group B* Streptococcus* was treated successfully with IR guided drainage and antibiotics.* Conclusion*. Though rare, GBS can still be a cause of invasive infection even in individuals who are nonpregnant and have no underlying comorbidities. Empiric antibiotic coverage for this organism should be kept in mind when treating an abscess.

## 1. Introduction

Invasive Group B* Streptococcus* (GBS), a leading cause of illness and death among infants in the first week of life and of infection in pregnant women, also causes significant morbidity and mortality among nonpregnant adults [1]. GBS has been traditionally considered a neonatal pathogen; however due to effective screening and intrapartum chemoprophylaxis the incidence of the disease has drastically fallen among this population [1, 2].

Recently GBS has emerged as an important cause of invasive infection in nonpregnant adults. In a large, population-based analysis the incidence of adult GBS disease has more than doubled over an observed 18-year study period from 3.6 cases per 100,000 persons in 1990 to 7.3 cases per 100,000 in 2007 [3]. Typical presentations of GBS disease in adults include bacteremia without focus and skin and soft-tissue infection. Other serious clinical syndromes, such as meningitis, pneumonia, streptococcal toxic shock syndrome, and endocarditis, are rare but are often associated with considerable morbidity and mortality [1, 4–11]. Nonfocal bacteremia and pneumonia were more common among patients aged 65 years and older. Osteomyelitis, skin and/or soft-tissue infection, peritonitis, meningitis, and necrotizing fasciitis were more common among younger adults [3]. Among adults, the most common risk factors for invasive GBS disease are older age and medical comorbidities; 88% of cases occur among persons with one or more underlying chronic medical conditions, especially diabetes [3–6, 8–10]. We highlight a rare case of GBS pelvic abscess in a nonpregnant patient with no significant comorbidities.

## 2. Case

A 43-year-old G2P1011 with a history of total vaginal hysterectomy six years before presentation, laparoscopic ovarian cystectomy, and a remote history of pelvic inflammatory disease presented to our emergency department with four days of pelvic pain, low-grade fever, chills, anorexia, and body aches.

**Figure 1:**
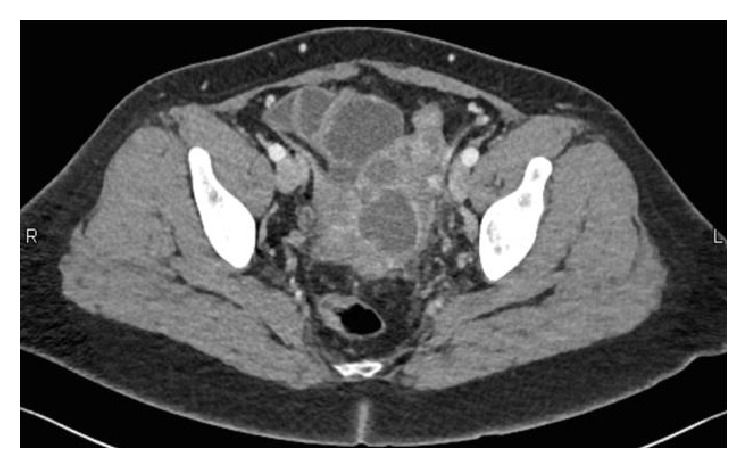
In the expected region of the uterus, there is a multiloculated cystic mass with enhancing walls concerning for pelvic abscess. This mass measures approximately 7.9 × 4.5 cm and is slightly to the left of midline.

On admission, the patient was febrile to 38.4°C and found to have leukocytosis (white count 14,900/mm^3^) with a left shift. While the patient was in the ED a pelvic ultrasound was obtained and was significant for a large (7.0 × 5.2 × 6.9 cm) heterogeneous complex lesion with multiple septations in the left adnexa with apparent fallopian tube involvement concerning for tuboovarian abscess. The decision was made to admit the patient. A pelvic CT with contrast was completed (Figure 1) and found a multiloculated cystic mass measuring 7.9 cm with enhancing walls in the pelvis diagnostic of a pelvic abscess. No noted abnormalities were seen in the small bowel or colon.

**Figure 2:**
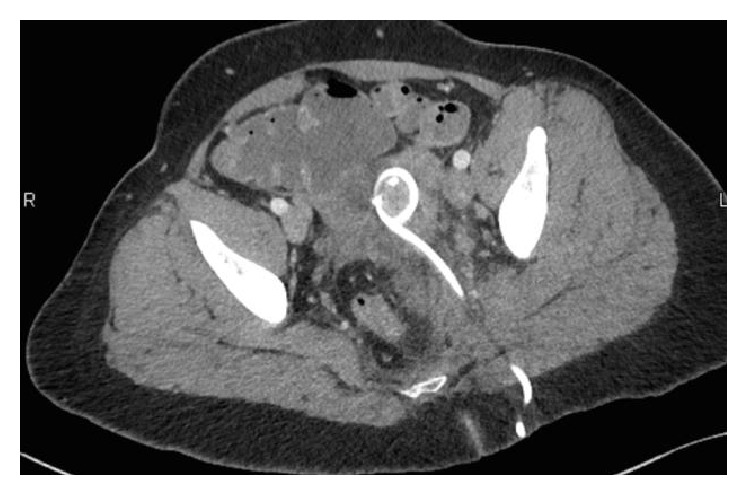
Ultrasound, fluoroscopic, and cone beam CT guidance; a 21-gauge needle was advanced into the fluid collection via a posterior transgluteal approach. A Greb set was used to secure access. An 8.5-French Cook all-purpose drainage catheter was placed over a wire into the collection and locked in place. Approximately 10 cc of purulent fluid was obtained and sent to the lab for analysis.

After blood cultures had been drawn in the ED, which yielded no growth at five days, the patient was empirically started on IV Cefoxitin, Doxycycline, and Flagyl and referred to our interventional radiology department for possible drainage. On day 1 of admission, an 8.5-French Cook all-purpose drainage catheter was placed transgluteally within the pelvic abscess (Figure 2). After placement, the Cook catheter drained purulent material that was subsequently sent for culture. The specimen showed Gram-positive cocci that speciated to GBS, resistant to clindamycin and erythromycin.

**Figure 3:**
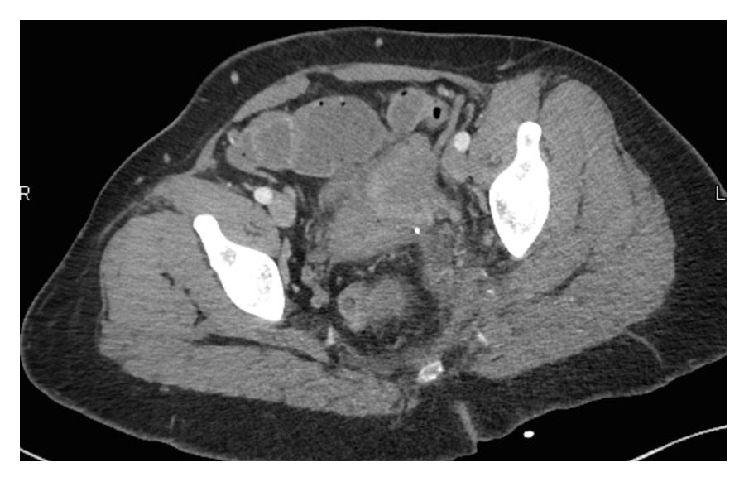
There has been interval improvement of the pelvic abscess with small residual locules of fluid adjacent to the drainage catheter measuring 3.5 cm in AP diameter. There are no foci of air within the residual locules.

Over the course of the admission the patient clinically improved, she remained afebrile, leukocytosis resolved to normal, and drainage decreased gradually. Four days after the catheter placement an interval CT showed a marked improvement of the pelvic abscess with only small residual locules of fluid found adjacent to the drainage catheter. At this stage, the interventional radiology team removed the catheter with no noted complications (Figure 3).

The patient remained afebrile with resolved leukocytosis and was discharged home on a course of oral antibiotics with follow-up in two weeks.

## 3. Discussion

Upon thorough review of the literature, all large studies describing GBS disease burden among multiple populations fail to mention GBS as a causative microorganism of deep-seeded pelvic abscesses. A search of the English literature using PubMed was conducted; cases were included if the patient was a nonpregnant adult with an abscess occurring in the pelvis or abdominal cavity. To the best of our knowledge, this is the first case of a sporadic pelvic abscess of unknown origin in a previously healthy patient with no medical comorbidities.

Published cases of deep-seeded GBS abdominal or pelvic abscesses were reported in patients with previous comorbidities, mostly diabetes, and involved an infection of the kidney [12–14], bladder [15], prostate [16], adrenal gland [17], liver [18], and uterus [19]. One case of sporadic GBS abdominal abscess was recently described in 2015 [20].

The most common cause of pelvic abscesses is fistula formation. The majority of cutaneous fistulas represent a complication of recent abdominal surgery [21]. The leading causes of internal fistulas are Crohn's disease, diverticulitis, malignancy, or a complication of treatment of these entities [22–26]. Other causes of pelvic abscesses include tuboovarian abscess (TOA) and complication following surgery for a ruptured appendix.

The predominant bacteria in intra-abdominal and pelvic abscesses with an intestinal culprit are polymicrobial in origin and the most common isolates in most series are the* B. fragilis* group and* E. coli* [27, 28]. Pelvic abscesses caused by TOAs are predominantly also polymicrobial in nature, although* Neisseria gonorrhoeae* and* Chlamydia trachomatis* are occasionally cultured in sexually active women of the reproductive age [29–31].

Overall, this case we present is unusual for numerous reasons. The monobacterial nature of the pelvic abscess, the deep-seeded pelvic site, the absence of an ascending route given the history of total vaginal hysterectomy with an intact vaginal cuff, and the lack of medical comorbidities in this patient make this finding extremely unlikely.

As the number of GBS infections is increasing yearly, clinicians are becoming more prone to include GBS as a culprit in their differential diagnoses for patients with multiple comorbidities, especially diabetes. This case, however, shows the ability of GBS to cause severe and invasive disease in a previously immunocompetent patient with no risk factors. Given the wide range of clinical presentations and capacity to cause invasive disease in healthy patients, clinicians should consider GBS in their differential, especially in sporadic abdominal or pelvic abscesses.
